# Primary diffuse large B-cell lymphoma of the CNS, with a “Lymphomatosis cerebri” pattern

**DOI:** 10.4322/acr.2021.250

**Published:** 2021-04-23

**Authors:** Balamurugan Thirunavukkarasu, Kirti Gupta, Ritu Shree, Anuj Prabhakar, Aastha Takkar Kapila, Vivek Lal, Bishan Radotra

**Affiliations:** 1 Post Graduate Institute of Medical Education and Research, Department of Histopathology, Chandigarh, India; 2 Post Graduate Institute of Medical Education and Research, Department of Adult Neurology, Chandigarh. India; 3 Post Graduate Institute of Medical Education and Research, Department of Radiodiagnosis, Chandigarh. India

**Keywords:** s, Central Nervous System Neoplasm, Lymphomatosis cerebri, Non-Hodgkin lymphoma, Dementia

## Abstract

We describe an unusual case of lymphomatosis cerebri in a middle-aged lady presenting with rapid-onset dementia. The lymphomatous infiltrate, instead of forming mass lesions, percolated throughout the brain parenchyma, which is often missed on a stereotactic biopsy and hence warrants caution and awareness about this entity. The nonspecific symptoms at presentation and a variable picture at imaging make this entity diagnostically challenging.

## INTRODUCTION

Primary central nervous system lymphoma (PCNSL) is a rare form of non-Hodgkin lymphoma accounting for 2% of primary tumors of CNS.^1^ It is confined to the brain, spinal cord, and eyes without systemic involvement. The majority of PCNSL are composed of diffuse large B-cell lymphoma (DLBCL) (90-95%) followed by Burkitt lymphoma (<5%), lymphoblastic lymphoma (<5%), marginal zone lymphoma (<3%), and T-cell lymphoma (2-3%).^2^ ^,^ ^3^ PCNSL usually presents as a well-demarcated, unifocal, or multifocal lesion in the brain. A rare morphological variant of PCNSL is lymphomatosis cerebri (LC). Drawing an analogy from gliomatosis cerebri, Bakshi et al.^4^ coined the term ‘lymphomatosis cerebri’ for this unique pattern of PCNSL wherein the atypical lymphoid cells strut across the entire brain parenchyma without causing any cohesive mass. LC has been described in anecdotal case reports.^5^ ^,^ ^6^ Its clinical presentation as rapidly progressive dementia with heterogenous imaging features makes this entity diagnostically challenging. Moreover, the subtle passive infiltration of the brain parenchyma by atypical lymphoid cells mandates caution when evaluating small biopsies.

## CASE REPORT

A 48-year-old immunocompetent female patient presented with a subacute (two months history of new-onset seizures) history of generalized seizures followed by right hemiparesis, headache, and vomiting. Her weakness improved completely after she received steroids at an outside center; however, her condition deteriorated rapidly over the next 2 weeks to altered sensorium, and she was referred to our institute for further evaluation. At the presentation, she was stuporous. There was spontaneous eye-opening; however, she was not responding to commands. There was a paucity of movements and hyperreflexia in the right upper and lower limbs, plantar withdrawal response on the right side, and extensor on the left side. The Glasgow coma scale was 10, and the modified Rankin scale was 5. The gadolinium-enhanced magnetic resonance imaging (MRI) of the brain suggested multifocal areas of altered signal intensities involving cortical as well as deep gray matter, subcortical and deep white matter, as well as the corpus callosum, with patchy areas of enhancement, minimal mass effect, and focal areas of diffusion restriction. There was no evidence of hemorrhage (Figures 11D). The HIV serology was negative.

**Figure 1:**
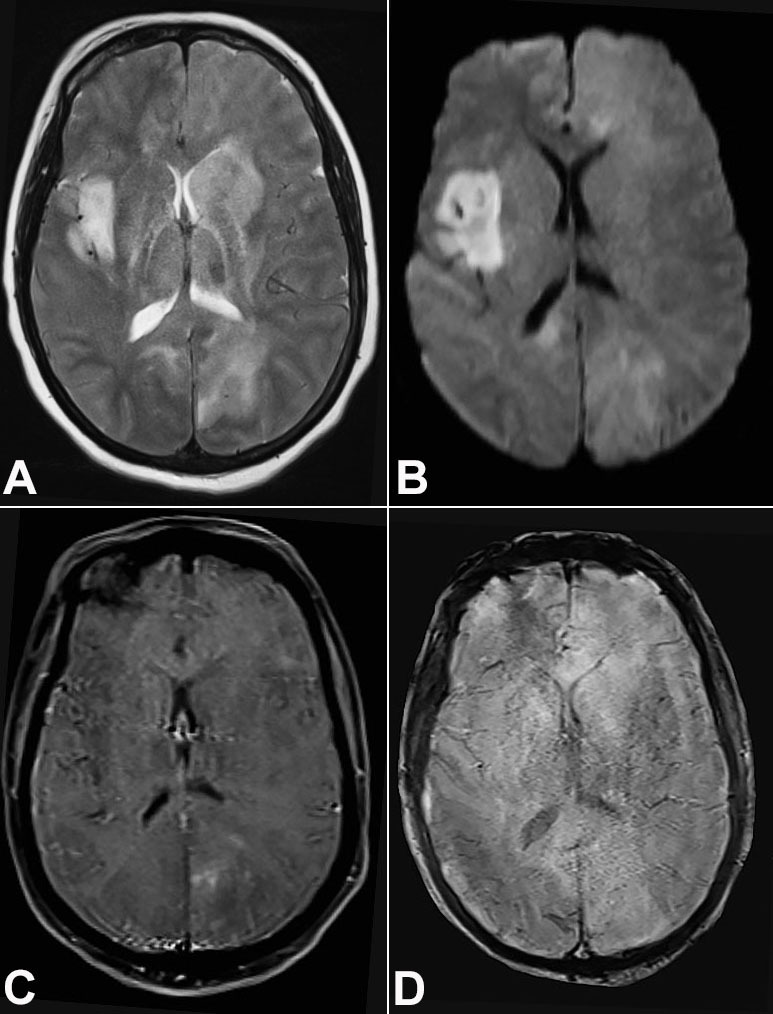
Brian MRI. **A –** Axial T2 weighted image shows ill-defined hyperintense signal involving bilateral basal ganglia (Left > Right), thalami, right insula, cortical and subcortical white matter in bilateral occipital lobes (Left> Right); **B –** Axial Diffusion weighted image shows areas of patchy diffusion restriction, more in the right insula; **C –** Axial T1 weighted post contrast image shows areas of mild patchy left occipital enhancement; **D –** Axial SWI image shows no evidence of hemorrhage

The cerebrospinal fluid (CSF) analysis (Table 1) revealed lymphocytic pleocytosis along with normal protein and glucose levels.

**Table 1:** Cerebral spinal fluid examination

Protein (mg/dl)	33	15-60
Glucose (mg/dl)	51	45-80
Cytology	Lymphocytic pleocytosis	-
Gram stain	Negative	-
Culture	Sterile	-
India ink	Negative	-
Cryptococcal antigen	Negative	-
Ziehl-Neelsen stain	Negative	-
HSV PCR	Negative	-

HSV PCR: Herpes simplex virus polymerase chain reaction

The laboratory workup is shown in Table 2.

**Table 2:** laboratory work up

**Test**	**result**	**RR**	**Test**	**result**	**RR**
Hb (g/dl)	10.9	12-15.5	Ca+ (mg/dl)	8.8	8.8-10.2
RBC (10^12^/L)	3.5	4.0-5.2	PO4 (mg/dl)	2.8	2.7-4.5
Ht (%)	34.2	36-46	Mg+ (mg/dl)	1.6	1.58-2.55
RDW (%)	18.2	11-14.5	AST (U/L)	21	2-40
MCV	81.6	80-100	ALT (U/L)	10	2-41
MCHC	32	31-37	ALP (U/L)	80	42-128
TLC (/mm^3^)	18000	4000-11000	T bil (mg/dl)	1.2	0.2-1.2
Plt (x10^5^/m)	2,51	1,5 -4,5	D bil (mg/dl)	0.3	0-0.3
ESR (mm - 1^st^ hour)	11	2-20	total protein (g/dl)	5.7	6.4-8.3
CRP (mg/L)	24.69	<10	albumin (g/dl)	3.3	3.4-4.8
Creatinine (mg/dl)	0.7	0.5-1.2	uric acid (mg/dl)	4.1	3.5-7.0
Urea (mg/dl)	13	10-50	LDH (U/L)	319	135-225
Sodium (mEq/L)	135	135-145	procalcitonin (ng/ml)	0.17	0.01-0.50
Potassium (mEq/L)	3.6	3.5-5.0	Ammonia (µmol/L)	44.5	19-71
Chloride (mEq/L)	101	90-107	HbsAg/ Anti HCV/ HIV	Non-reactive	-

ALP: Alkaline phosphatase, ALT: Alanine transaminase, AST: Aspartate transaminase, CRP: C-reactive protein, ESR: Erythrocyte sedimentation rate, Hb: Hemoglobin, RBC: HCT: Hematocrit, HCV: Hepatitis C virus, HbsAg: Hepatitis B surface antigen, HIV: Human immunodeficiency virus, LDH: Lactate dehydrogenase, MCH: Mean corpuscular hemoglobin, MCHC: Mean corpuscular hemoglobin concentration, MCV: Mean corpuscular volume, Ptl= platelets, RDW: Red blood cells, Red cell distribution width, TLC: Total leucocyte count, T bil: total bilirubin, D bil: direct bilirubin.

The Chest X-ray was normal, as well as the abdominal ultrasonography. The contrast-enhance computed tomography showed a well-defined homogeneous thin-walled cyst (2.5x 2.7x 3.4 cm) in the left ovary, likely a functional cyst. The PET-CT discarded any extracerebral or systemic lymphoma. On repeating imaging, a week later, there was a gross increase in the lesions with supratentorial herniation. She deteriorated rapidly and succumbed to her illness after five days of hospital stay. Due to rapidly progressive disease, possibilities of malignancy (lymphoma versus diffuse glioma), fulminant demyelination, fungal or parasitic infection were kept, and a brain autopsy was performed.

The brain, at autopsy, weighed 1300 g (mean Reference range 1240 g). There were no exudates on the convexities of the brain base. The leptomeninges were translucent. There were mild uncal notching and tonsillar herniation, indicating cerebral edema. Coronal slices revealed focal prominence of the intraparenchymal vessels in all the slices. No focal lesions were identified. The striate cortex showed softening and irregular grey-white junction bilaterally (Figures 22C).

**Figure 2:**
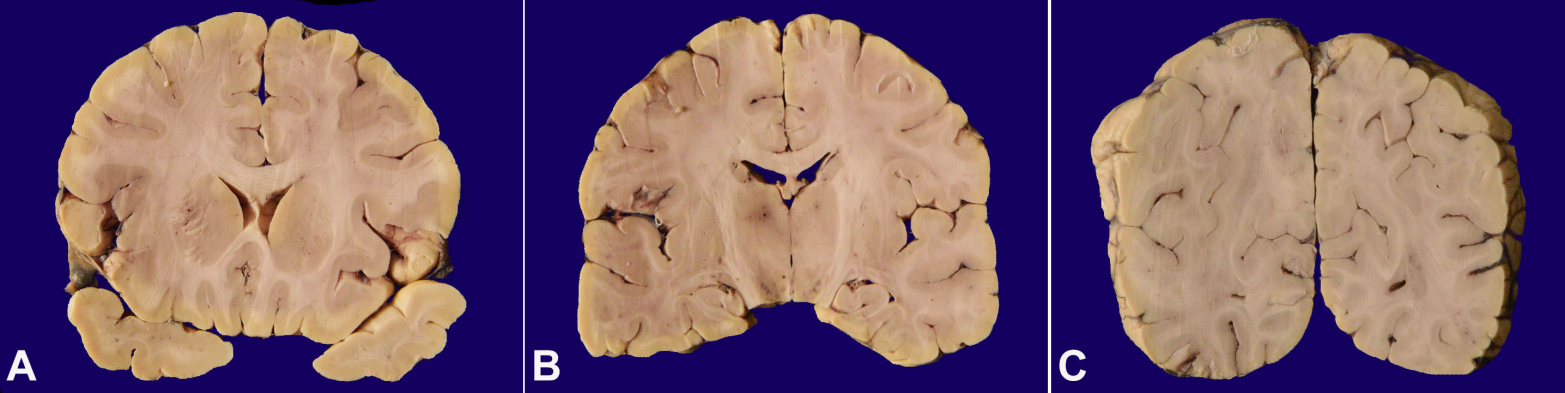
Gross view of the brain. **A –** Coronal section showing irregularity in left anterior limb of internal capsule extending to head of caudate; **B –** Prominence of intraparenchymal vessels in bilateral thalamus; **C –** Loss of grey-white junction noted in left striate cortex with surrounding edema.

**Figure 3:**
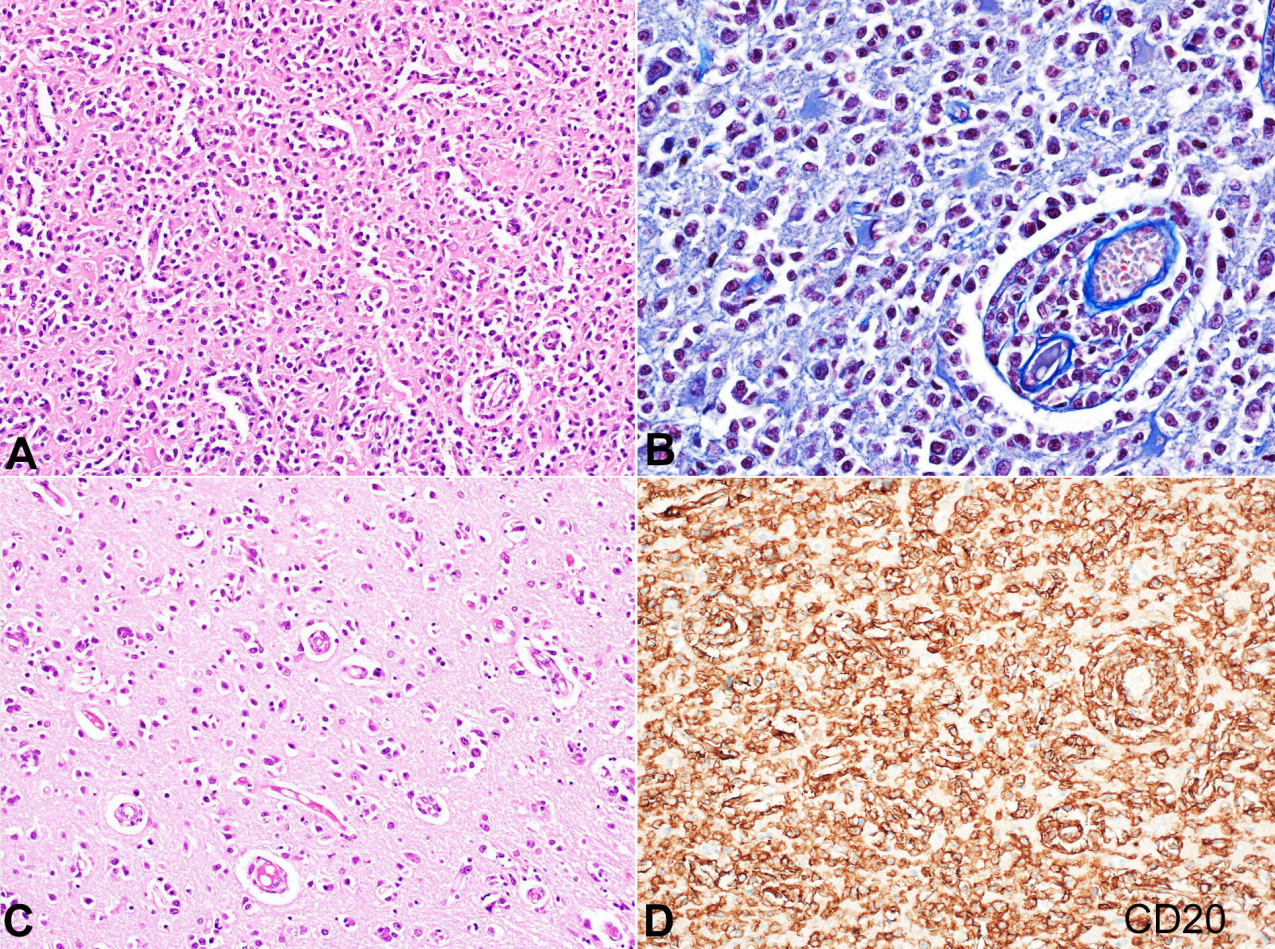
Photomicrographs of the brain. **A –** Low magnification of striate cortex depicting non-cohesive, atypical lymphoid cells percolating through the parenchyma (H&E x200); **B –** Focal areas showing a modest perivascular cuffing with finely dispersed lymphoma cells in adjacent parenchyma (Martius scarlet blue x400); **C –** High magnification of frontal cortex depicting fine infiltration by lymphoma cells (H&E x400); **D –** Strong diffuse immunopositivity of neoplastic cells with CD20 (immunoperoxidase x400).

The brain stem, cerebellum, and upper part of the cervical cord appeared normal on gross examination. Microscopic sections from the striate cortex revealed the maximum burden of the disease with a subarachnoid collection of atypical lymphocytes, characterized by round to oval in shape and twice the size of mature lymphocytes. Nucleolar prominence was noted occasionally. These were seen percolating along the entire grey matter and white matter without forming mass lesions (Figures 33B). There was a modest tendency to aggregate around the vessels. The remaining frontal cortex, cingulate gyrus, amygdala, hippocampus, central white matter, basal ganglia showed a very scant and delicate infiltrate of similar cells strutting through the parenchyma characteristic of LC pattern noted in primary CNS Diffuse large B cell lymphoma. (Figure 3C). Neoplastic cells were positive for CD20 (Figure 3D), MUM1, Ki 67 of 70%, while negative for CD10, Bcl6, CD138. CD3 and CD68 highlighted very few T lymphocytes and macrophages. EBER-ISH was negative. Subcortical areas showed patchy myelin pallor without over demyelination secondary to infiltration by the lymphoma cells.

## DISCUSSION

Lymphomatosis cerebri is a rare histological pattern of PCNSL with less than 20 cases in immunocompetent patients reported as single case reports in the literature.^4^ ^-^ ^11^ Due to its rarity, the exact incidence is not known. In majority, this pattern has been described with B cell lineage, with tumor cells expressing CD19, CD20, and CD79a, few rare examples of T cell Is also on record.^12^ Patients mostly present with variable clinical symptoms such as gait disturbance, focal weakness, decline of cognitive function, memory disturbance, personality changes, dementia, anorexia, orthostatic hypotension, paraparesis, and weight loss.^10^ Importantly, the clinical presentation of LC is often confounded, as the symptoms like dementia and personality change are often not noticeable and attributed to increasing age. Moreover, such symptoms are easily mistaken for other more common conditions such as infectious, inflammatory, vascular, toxic, or neurodegenerative etiologies causing white-matter damage. To further complicate things, the treatment with corticosteroids gives temporary symptomatic relief and may obscure the biopsy findings.^6^ In the past two decades, an increased incidence of PCNSL has been reported in patients older than 60 years. However, it can affect patients of any age, with a peak incidence during the fifth to seventh decades of life.^13^ Useful diagnostic histologic clues include the atypical nature of the discrete lymphoid cells, mitotic figures, and apoptotic bodies that are often deceptively admixed with the atypical cells and should be carefully searched for, especially in a stereotactic biopsy.

At gross, despite the absence of any mass-like lesions, they produce subtle changes of loss of grey-white distinction, the granularity of the parenchyma, and edema.^5^ On imaging, primary CNS lymphomas present as homogeneously enhancing periventricular or superficial lesions that are iso to hypodense on CT, iso or hypointense on T1 and hypointense to grey matter on T2.^14^ Lymphomatosis cerebri, on the other hand, presents as diffuse and asymmetric, abnormal T2-hyperintensity in deep and subcortical white matter with involvement of corpus callosum. Contrast-enhancement pattern may vary from no-enhancement to patchy non-mass-like enhancement or nodular/mass-like enhancement^.^.^15^ Intravascular lymphoma (IL) is an important differential diagnosis on imaging and may presents with infarct-like lesions, nonspecific diffuse white matter lesions, meningeal enhancement, or large mass-like lesions.^16^ It needs mention and differentiation from LC; the neoplastic cells in this entity (IL) selectively grow within the lumens of small vessels and capillaries and do not form mass lesions. This pattern is likely caused by a defect in the homing receptors in the neoplastic cells.^17^ Other differentials include diffuse glioma (gliomatosis cerebri), progressive multifocal leukoencephalopathy (PMLE), vasculitis, microangiopathy, and demyelination.^10^ The diffuse infiltration of the brain parenchyma evident in gliomatosis Is a potential diagnostic pitfall; however, immunoreactivity for glial fibrillary acidic protein (GFAP) distinguishes the former from the latter. Likewise, the splitting of argyrophilic fibers around the vessels in case of LC and PCNSL, brings vasculitis and microangiopathy amongst the differentials. The neoplastic nature of the infiltrate within the vessels helps in resolving the diagnostic dilemma. Demyelination noted in cases of PMLE typically affects the white matter anywhere within the central nervous system. It is caused due to the JC virus, the inclusions of which are evident in the oligodendrocytes as ground-glass nuclei, and reveals positivity with SV40 immunohistochemistry.^18^

Interestingly, studies have indicated variations in the expression of Lymphocyte Function Associated Antigen 1 (LFA-1 or CD11a, α1β2), which may be responsible for differences in individual tumor cell permeation of the brain as these discohesive, infiltrating cells in LC have been detected to show less immunostaining for LFA-1 than did lymphoma cells within compact cell clusters.^17^

In conclusion, this summary features an unusual variant of PCNSL, which may often be missed on stereotactic biopsy owing to the nonspecific symptoms at presentation, the variable picture at imaging, and subtle infiltrative nature of the lesion at histology. Furthermore, despite the poor outcome, intensive treatment may be considered, especially in patients with better Karnofsky Performance Status.

## References

[1] Ostrom QT, Gittleman H, Fulop J, Liu M, Blanda R, Kromer C, Wolinsky Y, Kruchko C, Barnholtz-Sloan JS (2015). CBTRUS Statistical Report: Primary Brain and Central Nervous System Tumors Diagnosed in the United States in 2008-2012. Neuro Oncol..

[2] Ferreri AJ, Marturano E (2012). Primary CNS lymphoma. Best Pract Res Clin Haematol.

[3] Chihara D, Fowler NH, Oki Y (2018). Impact of histologic subtypes and treatment modality among patients with primary central nervous system lymphoma: a SEER database analysis. Oncotarget.

[4] Bakshi R, Mazziotta JC, Mischel PS, Jahan R, Seligson DB, Vinters HV (1999). Lymphomatosis cerebri presenting as a rapidly progressive dementia: Clinical, neuroimaging and pathologic findings. Dement Geriatr Cogn Disord.

[5] Gupta K, Gupta V, Radotra BD, Tewari MK (2019). “Slow and Steady” infiltrates the brain: an autopsy report of Lymphomatosis Cerebri. Neurol India.

[6] Sugie M, Ishihara K, Kato H, Nakano I, Kawamura M (2009). Primary central nervous system lymphoma initially mimicking lymphomatosis cerebri: an autopsy case report. Neuropathology.

[7] Carlson BA (1996). Rapidly progressive dementia caused by nonenhancing primary lymphoma of the central nervous system. AJNR Am J Neuroradiol.

[8] de Toledo M, López-Valdés E, Ferreiro M (2008). Linfomatosis cerebral como causa de leucoencefalopatía. Rev Neurol.

[9] Keswani A, Bigio E, Grimm S (2012). Lymphomatosis cerebri presenting with orthostatic hypotension, anorexia, and paraparesis. J Neurooncol.

[10] Kitai R, Hashimoto N, Yamate K (2012). Lymphomatosis cerebri: clinical characteristics, neuroimaging, and pathological findings. Brain Tumor Pathol.

[11] Leschziner G, Rudge P, Lucas S, Andrews T (2011). Lymphomatosis cerebri presenting as a rapidly progressive dementia with a high methylmalonic acid. J Neurol.

[12] Kim EY, Kim SS (2005). Magnetic resonance findings of primary central nervous system T-cell lymphoma in immunocompetent patients. Acta Radiol.

[13] Villano JL, Koshy M, Shaikh H, Dolecek TA, McCarthy BJ (2011). Age, gender, and racial differences in incidence and survival in primary CNS lymphoma. Br J Cancer.

[14] Haldorsen IS, Espeland A, Larsson EM (2011). Central nervous system lymphoma: characteristic findings on traditional and advanced imaging. AJNR Am J Neuroradiol.

[15] Izquierdo C, Velasco R, Vidal N (2016). Lymphomatosis cerebri: A rare form of primary central nervous system lymphoma. Analysis of 7 cases and systematic review of the literature. Neuro-oncol.

[16] Yamamoto A, Kikuchi Y, Homma K, O’uchi T, Furui S (2012). Characteristics of Intravascular Large B-Cell Lymphoma on Cerebral MR Imaging. AJNR Am J Neuroradiol.

[17] Paulus W, Jellinger K (1993). Comparison of integrin adhesion molecules expressed by primary brain lymphomas and nodal lymphomas. Acta Neuropathol.

[18] Sugita Y, Muta H, Ohshima K (2016). Primary central nervous system lymphomas and related diseases: pathological characteristics and discussion of the differential diagnosis. Neuropathology.

